# Supplementary material for: The unnatural history of Kāne‘ohe Bay: coral reef resilience in the face of centuries of anthropogenic impacts
Source: PeerJ. 2015 May 12;3:e950. doi: 10.7717/peerj.950 (PMC4435448; doi:10.7717/peerj.950)
Supplement: Supplemental Information 1 — Extensive detail of the description of the features, physical, biological and chemical characteristics of Kāneʻohe Bay. [file peerj-03-950-s001.docx]

**Kāneʻohe Bay Ecosystem**

*Description*

Twenty five million years ago, the Waiʻanae and the Koʻolau volcanoes formed Oʻahu. A massive earthquake caused the windward side of the Koʻolau volcano break away causing a landslide into the Pacific Ocean over one million years ago. The debris from the Nuʻuanu avalanche formed the northern and western boundaries of Kāneʻohe Bay  (Jokiel, 1991). Kāne‘ohe Bay was further facilitated by erosion via rivers, changing sea level, and sediment deposition  (Jokiel, 1991). Kāne‘ohe Bay ecosystem is composed of different geographic features including a large estuarine bay, the adjacent watershed, a protecting barrier reef that contains a sandbar (Ahu o Laka), and the near shore oceanic environment  (Jokiel, 1991). Two navigable channels cross the barrier reef for entrance into the bay. The Sampan Channel (Kāne‘ohe Passage) has a natural depth of 2.4 m and allows access at the southeast portion of the bay for smaller ships. This channel intersects a deeper dredged ship channel 1.5 km west of Mokapu, which extends the entire length of the bay  (Devaney et al., 1982). At the end of the ship channel is the Mokoliʻi passage, which was dredged to 7.6 m to allow access for larger ships between the bay and the open ocean (Jokiel, 1991). Additionally, the bay holds four major islands and islets including Kapapa, Mokoli‘i (Chinaman’s Hat), Kekepa (Turtleback Rock), and Moku o Lo‘e (Coconut Island). Located in the inshore area, Moku o Lo‘e and Mokoli‘i are the only basaltic islands in the bay, which are remnants of the eroded volcano series. On the barrier reef near the Mokapu Peninsula, the islets Kapapa and Kekepa are submerged lithified dunes that have changed to stone and are currently being eroded by wave action (Jokiel, 1991).

*Islets*

Comprised of basalt, limestone and sand, Kapapa Island rises only a few feet above sea level, which was formed by coral accretion from high sea level (+1.5m) during the 5500-2000 year meltwater pulse. Though this isle is only three acres, it is an important nesting site for the Bulwer's Petrel (*Bulweria bulwerii*, ‘ou) and over 500 Wedge-tailed Shearwaters (*Puffinus pacificus*, ua‘u kani) that nest in burrows underground between June and October. Native flora can be found growing on Kapapa interspersed with introduced ironwood trees. The rich archeological history of Kapapa was unveiled with the discovery of an ancient Hawaiian fishing shrine (koa), a religious temple (heiau), as well as middens and burial sites dating from 1750 AD; thereby securing a place for the island on the National Registry of Historical Places.

The largest island in the bay, Moku o Loʻe, is a remnant of the basalt ridge that formed the Pohakea headland during the volcanic activity of the Ko‘olau volcano. This island is located in the southwest portion of the bay and was originally the home of Hawaiian ali‘i (royalty), including Kamehameha I and Bernice Pauahi Bishop. Later in 1844, the royal lu‘au (feast) for Queen Emma was held on Moku o Lo‘e (Devaney et al., 1982). Traditionally, Moku o Lo‘e served as a way station for fishing, where Native Hawaiians and later commercial fishermen would mend their nets and repair their boats. In 1933, Christian R. Holmes, who was the heir to the Fleischman yeast fortune and owner of Hawaiian Tuna Packers (now Coral Tuna), purchased Moku o Lo‘e. Holmes transformed the island into his own private paradise by extensively dredging and expanding the original size of the island from 0.05 km^2^ to 0.11 km^2^. In 1944, the island became the Army Rest and Recreation Center for war-weary servicemen, until in 1947, when five wealthy oilmen purchased the island with the intention to develop a hotel and country club. The island became a summer retreat for one of these men, Edwin Pauley, who bought out the other partners and became sole owner of the island. Pauley donated much of the shoreline of the Moku o Loʻe to the University of Hawaiʻi to establish the Hawai‘i Marine Lab in 1947 (Smith et al., 1981). Later in 1965, the Hawai‘i Marine Lab was changed to the Hawai‘i Institute of Marine Biology. After the death of Edwin Pauley, the private portion of the island was sold to a Japanese real estate developer, Katsuhiro Kawaguchi. The private sector of the island was later purchased back from Mr. Kawaguchi with funds provided to the University of Hawai‘i by the Pauley Family in 1995. The generous history of the Pauley Family to researchers at the University of Hawai‘i has facilitated the extensive investigation of Kāne‘ohe Bay (Smith et al., 1973; Banner, 1974; Devaney et al., 1982; Holthus, 1986; Jokiel, 1991; Hunter & Evans, 1995). Kāne‘ohe Bay has an intriguing history of anthropogenic influence from early Polynesian settlement through post European contact, and is arguably the most intensively studied coral reef ecosystem in the world with over 1,600 publications to date (Kāneʻohe Bay Information System, 2014).

**Hydrology**

The Kāneʻohe Bay watershed is 97 km^2^ and 60% of the watershed is located in the coastal lowlands. In the watershed, rainfall averages about 200 cm yr^-1^ and ranges between 85 cm yr^-1^ to 365 cm yr^-1^ (Giambelluca et al., 1986; Drupp et al., 2011). In 1916, the Waiāhole ditch tunnel system diverted water from the windward side to the leeward side of the island of Oʻahu for large-scale farming on sugar cane plantations (Takasaki et al., 1969). This ditch system resulted in decreased stream flow, dried springs, and reduced groundwater storage (Devaney et al., 1982). Stream runoff in the bay was documented to decrease by 40%, from an estimated 3.7 m^3^ d^-1^ to 2.1 m^3^ d^-1^ (Maragos & Chave, 1973). Currently, eleven streams supply the bay with freshwater. Kāne‘ohe Stream is considered the most important freshwater source as it accounts for more than 75% of the discharge into the southern sector of the bay (Drupp et al., 2011). This stream drains an area of highly developed and populated land transporting anthropogenic and terrigenous materials into the bay. The mean discharge of Kāneʻohe Stream is 0.20 m^3^ s^-1^ and peaks greater than 0.45 m^3^ s^-1^ after high precipitation  (Ostrander et al., 2007). The stream enters the bay through a small manmade channel (<15m width) (Hoover, 2004) and creates a plume with a surface area of 9.9 km^2^ (Ostrander et al., 2007). The persistence of a freshwater plume in the south bay is controlled by the discharge volume and duration of the stream, although spatial variability of the plume is primarily determined by atmospheric forcing  (Ostrander et al., 2007).

Bathen  (Bathen) conducted a thirteen-month hydrographic survey that highlighted the importance of bathymetry on the patterns of water circulation in the bay. In addition to characterizing the hypsographic conditions, tides, circulation patterns, volume transports, sewage distribution, heat budget, precipitation, and runoff patterns, Bathen (1968) concluded that the reef components largely constrain the flow within the bay. Interestingly, the southern portion of the bay is somewhat isolated hydrologically from the rest of the bay (Bathen, 1968; Hearn & Atkinson, 2000; Lowe et al., 2009; Massaro et al., 2012).

**Bathymetry and Physiography**

The bathymetry of the bay is divided into the inshore and offshore portions (Bathen, 1968). The inshore portion comprises 66% of the total area with an average depth of 6.1 m and is characterized by the estuarine lagoon that holds numerous patch reefs (Jokiel, 1991). The shoreline, except parts of Mokapu Peninsula, is ringed by shallow fringing reef 0.3-0.9 m deep (Cox et al., 1973). The deepest portion of the bay is 19 m. The remaining 34% of the bay is offshore, which consists almost entirely of extensive shallow coral and sand reef 0.3 m-1.2 m in depth.

The bay is also divided into three physiographic cross sections (i.e. inshore, inner bay, and outer bay) that describe the ecosystem influence on the bathymetry, communities, flora and fauna observed within each section and the interplay among them. These physiographic regions were established based on bathymetry, water circulation, physiography, and oceanic influence (Bathen, 1968; Smith et al., 1981). The inshore consists of the intertidal zone along the shoreline and the fringing reef. The lagoon and patch reefs are located the inner bay zone. The lagoon is generally divided into sectors southeast, central and northwest based on the circulation and relative degree of oceanic influence (Bathen, 1968; Smith et al., 1981). Lastly, the outer bay section consists of the barrier reef and the two channels bisecting it (Jokiel, 1991).

**Environmental Conditions**

*Circulation Patterns*

Evaporation, precipitation, wind, and stream runoff all influence the environmental conditions in Kāneʻohe Bay (Bathen, 1968; Drupp et al., 2011). The bathymetry of the bay in combination with mixed tides and wind patterns drive the circulations patterns (Bathen, 1968; summarized in Smith et al. 1973; Jokiel, 1991). Mean tidal range is 68 cm d^-1^ with a maximum of 110 cm d^-1^ (Jokiel, 1991). Also, the tidal height in the bay is 3 - 8.5 cm greater than that of Honolulu tides, where the bathymetry of the bay accounts for ~3 cm difference and additional differences are due to added effects of winds. Kona winds decrease the daily tidal height, whereas trade winds can add as much as 5.5 cm to predicted tides (Jokiel, 1991). During most of the year, physical mixing is driven predominantly by trade winds and to a lesser degree by semidiurnal tides (Drupp et al., 2011). Low winds can result in a vertical gradient in water clarity and salinity, and allow density stratification when freshwater input is high. Also, wind-induced mixing resuspends sediments into the water column, thereby reducing light penetration within the bay (Smith et al., 1973).

Consequently, water circulation in the northern bay is strong but the circulation is weakened in the south bay. This pattern of circulation results in southern portion of the bay having a longer residence time (~8 to > 30 days) and therefore is more influenced by riverine input than are the central and northern portions of the bay (Lowe et al., 2009). Therefore, the southern portion of the bay is somewhat isolated hydrologically from the rest of the bay (Bathen, 1968; Hearn & Atkinson, 2000; Lowe et al., 2009; Massaro et al., 2012). Most importantly, these circulation patterns determine the distribution of temperature, salinity, oxygen and phosphate and therefore coral distribution and abundance in the bay (Bathen, 1968; Ostrander et al., 2007).

*Salinity, Temperature, Dissolved Oxygen*

Water quality in the bay is dependent on water circulation, runoff from land, heat exchange and rainfall (Bathen, 1968). Salinity is greatly influenced by freshwater runoff from land and ranged between 18 – 36 ppt (Bathen, 1968; Massaro et al., 2012). Temperatures in the bay are spatially and seasonally variable (Bathen, 1968). During the summer season, the bay water average 1.5 – 2°C warmer then temperatures observed in the open ocean. Also during the summer months, temperature stratification in the bay increases and the thermocline (1 – 2 m) weakens (Bathen, 1968). Temperatures are most variable in deep inshore areas, while the highest temperatures can be observed in the shallow reef areas (Jokiel, 1991). Mean water temperatures range between 19.5 °C to 30 °C with an overall winter average of 25.6°C and a summer average of 27.4°C  (Jokiel, 1991; Massaro et al., 2012). Dissolved oxygen concentrations vary among water depth and location in Kāneʻohe Bay. In the upper three meters, dissolved oxygen concentrations average about 4.5 mL L^-1^. Deeper depths (3-8 m) average around 4.3 mL L^-1^, while waters below 8 meters have an average of 3.9 mL L^-1^ of dissolved oxygen. Dissolved oxygen saturation of surface waters (0-3 m) ranges between ~80% in early morning and ~120% in the late afternoon (Drupp et al., 2011). Also, high concentrations of dissolved oxygen are observed in the channels of the bay (Jokiel, 1991). Dissolved oxygen concentration also varies with the direction of the tide. Falling tides show a greater increase in oxygen and pH over rising tides (Klim, 1969). Also, oxygen concentration correlations with changes in pH, suggesting that photosynthesis and respiration are stronger drivers of pH and dissolved oxygen changes  (Klim, 1969). Likewise, in a recent modeling exercise, Jury et al. (2013) find that biological activity dominates water chemistry for Kāne‘ohe Bay, indicating that understanding ecosystem feedbacks is critical to predicting future conditions.

*Carbon Dioxide*

The local climate forcing (i.e., wind characteristics and storm events) are important factors in controlling the coastal CO_2_-carbonic acid system dynamics and air-sea exchange of CO_2_ in Kāne‘ohe Bay (Fagan & Mackenzie, 2007). Kāne‘ohe Bay is considered a net source of *p*CO_2_ (-1.45 mol C m^-2^ year^-1^) to the atmosphere (on an annual or long term scale) because of the extensive calcification throughout the bay and the heterotrophy in the southern sector (Fagan & Mackenzie, 2007; Massaro et al., 2012). However, Kāneʻohe Bay was observed to have infrequent and short-term periods of CO_2_ sinks over a 2.5 year period (Drupp et al., 2011).

The average *p*CO_2_ for Kāne‘ohe Bay was observed to be 448 ± 52 μatm with a 112μatm range (over a 1 year cycle) with the highest concentration in summer (September 2007) and the lowest in winter (February 2007) (Massaro et al., 2012). A well-defined diel cycle in *p*CO_2_ was observed with a median diel variability of 34 μatm and a range from 7 to 204 μatm over a 24-hour period (Massaro et al., 2012). *p*CO2 levels reached their maximum values in the early morning and minimum values in the late afternoon (Drupp et al., 2011). Biological processes are largely responsible for diurnal and short-term variations in *p*CO_2_ in Kāne‘ohe Bay through biological consumption of CO_2_ via primary production and calcium carbonate (CaCO_3_) dissolution (Massaro et al., 2012). Conversely, release of CO_2_ dominates below the euphotic zone and occurs through biological processes of respiration and calcification (0.6 mole CO_2_ per mole CaCO_3_) (Gattuso et al., 1998; Shamberger et al., 2011). In Kāne‘ohe Bay, coral calcification was determined to be the main factor that influences the CO_2_ flux (Fagan & Mackenzie, 2007; Shamberger et al., 2011). Additionally, the physical processes in the bay influence the variations of *p*CO_2_ (Wollast, 2002)*. p*CO_2_ rises with increasing temperature, while increased precipitation from storm events augments stream and land runoff, bringing excess nutrients to the bay waters (Paquay et al., 2007; Drupp et al., 2011). These nutrients stimulate phytoplankton blooms that impact *p*CO_2_; however, other physical factors, such as wind speed, solar radiation, and turbidity, also influence the frequency and length of phytoplankton blooms. The interaction of phytoplankton blooms and nutrient loading strongly affect the carbon dioxide dynamics within the coastal coral reef environment, where large blooms can decrease the CO_2_ in the bay waters via photosynthesis and reverse the direction of air-sea fluxes of CO_2_ (Fagan & Mackenzie, 2007).

The observed infrequent and temporary switches between source to sink of CO_2_ in Kāneʻohe Bay may indicate that these coral reef ecosystems will likely become weaker sources of CO_2_ to the atmosphere under future climate scenarios (Massaro et al., 2012). Moreover, rates of bioerosion increase at reduced pH, and enhance the chemical and biological dissolution rates of the carbonate structures (Silbiger et al., 2014). Ultimately, whether these ecosystems become net sinks of CO_2_ causing enhanced acidification in the seawater, in turn lowering the rates of calcification is dependent on biological responses to future climate conditions (Jury et al., 2013).

**Habitats**

Kāne‘ohe Bay exhibits a wide diversity of benthic habitats. Among these are the upper, middle, and lower intertidal zones, which in Hawai‘i are dominated more by the physical action of waves than tides (Bird et al., 2013). The shorelines also contain sandy and rocky shores, mud flats, and mangrove swamps. In the subtidal zone, many organisms dwell in the various reefs areas on the patch, fringing and barrier reefs. Also, the lagoon floor, limestone and lithified sand dunes within the bay provide additional habitats. In total, about 25% of the more than 6500 currently described species of Hawaiian coral reef organisms are endemic (Fautin et al., 2010), and many of these are found among the diverse habitats of Kāne‘ohe Bay.

*Barrier reef*

Kāne‘ohe Bay is protected by a structural barrier reef, which is about 2 km wide and more than 5 km long, at the seaward margin of the lagoon (AECOS, 1981; Jokiel, 1991). This barrier reef is not composed entirely of limestone, but rather a ridgeline of basalt that is overlaid with calcium carbonate. The basaltic reef structure is similar in form and function to a barrier reef but has a different developmental history (Jokiel, 1991, 2008). Between the barrier reef and the intertidal zone lies the lagoon that includes patch reefs that contain rich coral and algal communities along with sand and sea grass beds (Jokiel, 2008). As with many Hawaiian coral reefs, algal biomass and diversity are high (Vroom & Braun, 2010; Vroom, 2011), but reefs throughout the lagoon are now dominated by alien invasive algal species (Smith et al., 2002). Likewise, the lagoon supports a large abundance of phytoplankton and zooplankton (Smith et al., 1981; Taguchi & Laws, 1987; Ringuet & Mackenzie, 2005) as well as an abundant range of pelagic fishes from planktivores to top carnivores (Jokiel, 1991; Hunter & Evans, 1995).

*Patch reefs*

Kāne‘ohe Bay holds 79 patch reefs and 25 of those patch reefs have been partially or entirely dredged (Jokiel, 1991). The patch reefs range between 21 m to 850 m in diameter and have a collective surface area of 2.02 by 106 m^2^ (Roy, 1970; Jokiel, 1991). Most are found in proximity to the two channels across the barrier reef, suggesting physical environment rather than benthic substrate, is the primary determinant of patch reef formation (Jokiel, 1991). At mean sea level, the patch reefs are less than a meter underwater with some as deep as 1.5 - 3 meters (Jokiel, 1991). These patch reefs formed during periods of sea level rise through the upward growth of coral (Roy, 1970; Jokiel, 1991). The youngest patch reefs in the bay are estimated to have begun growth 12,000 years ago with maximum reef growth 5-8,000 years ago (Fletcher & Jones, 1997). Changes in sea level during the last 12,000 years were some of the greatest changes over the last 100,000 years (Fletcher & Jones, 1997). For example, between 5,500-2,000 years ago the sea level as 1.6 m high than today, which is thought to lead to the formation of Kapapa island on the barrier reef. Growth of coral and coralline algae is more active on the edges of the patch reefs with the under slopes supporting high coral growth (Jokiel, 1991). The flat tops of the patch reefs support little to no coral growth, likely because they are often exposed during spring tides. As a result, the reef tops are covered with sand, rubble, and algal nodules.

*Fringing reefs*

Surrounding the land perimeter of Kāneʻohe Bay, except parts of Mokapu Peninsula, lay fringing reefs in shallow waters (< 1m), which extend outward for 300-700m (Cox et al., 1973). These fringing reefs have developed through the accumulation of skeletons of calcifying organisms overgrowing the underlying basalt. Moku o Loʻe is also surrounded by fringing reefs. Located between the intertidal and reef crest on the fringing and patch reefs are reef flats. This area of the reef is often exposed to extremes of environmental conditions (e.g. freshwater runoff, air exposure, sedimentation) (Jokiel, 1991; Bahr et al., 2015), sometimes resulting in localized mortality   (Jokiel et al., 1993). Substrata in this habitat consist of mud, silt, carbonate sand, coral rubble and limestone rock (Jokiel, 1991; Stimson et al., 2001), and may also contain coral mounds or ridges, communities of turf or fleshy algae, and small sea grass beds (Jokiel, 2008). Exposure to extreme environmental conditions (e.g. water motion, circulation, sedimentation, freshwater input) restricts coral abundance but high abundances of fleshy algae are broadly distributed within reef flats (Smith et al., 1981; Jokiel, 1991, 2008). The reef edge lies between the back and fore reef zones at the seaward edge of the reef flat, and is the highest part of the reef, which results in it often being exposed during low tides. Moving seaward past the reef edge is the reef slope where the coral cover, abundance and diversity of fishes are the highest (Jokiel, 1991).

*Mangroves*

Mangrove swamps are also found in Kāneʻohe Bay. Mangroves were first introduced to Hawai‘i in August of 1922 when 14,000 seedlings of four species of mangrove, two of which (*Rhizophora mangle* and *Bruguiera sexangula*) subsequently became established, were planted in the salt marshes of O‘ahu (Krauss & Allen, 2003). By 1963 mangroves covered 35 acres of the seaward edge of the He‘eia fishpond creating a mangrove swamp (Devaney et al., 1982). Current mangrove swamps are dominated by the red mangrove, *Rhizophora mangle*, but do not appear to be commonly used by native species, and instead appear to serve primarily as breeding and nursery areas for alien marine life in the bay (Demopoulos & Smith, 2010). The prop roots of the mangroves have altered the structure of these habitats by trapping sedimentary and organic detritus creating an area of deposition and decay. As a result, these areas accumulate a fine silty mud bottom that contains hydrogen sulfide and experience extreme fluctuations in physical parameters (e.g., dissolved oxygen, pH, salinity, temperature) due to high freshwater input from stream runoff during low tides and by seawater input from the bay during high tides (Jokiel, 1991). Furthermore, these areas take more than 3 decades to return to native condition upon removal of the alien mangroves due to the refractory nature of the mangrove roots and the lack of native consumers (Siple & Donahue, 2013).

**Corals in Kāneʻohe Bay**

*Distribution and Abundance*

Although the exact number remains a subject of some debate, Hawaiian waters contain about 40 coral species from eight families, of which only a few species are abundant (Maragos, 1972; Grigg, 1994; Forsman et al., 2009; Forsman et al., 2010). Historical anthropogenic influence of nutrient enrichment, dredging activities and land derived sediments have reduced coral abundance and diversity in the southern bay, and wave action and abrasion by suspended sediments reduce coral coverage in high-energy regions. Corals in Kāne‘ohe Bay are most commonly found on the reef crests and slopes of the fringing and patch reefs (summarized in Smith et al., 1973; Jokiel, 1991). The seaward portion of the barrier reef contains a lower percentage of live coral cover averaging to about 5-10%, most of which are high wave energy coral species such as *Pocillopora meandrina* and *Porites lobata*. These high wave energy species can also be found on some reefs near channels within the lagoon where surge and circulation conditions are strong (Maragos, 1972; Smith et al., 1973). While the seaward barrier reef is considered to have low coral cover, coral reefs located in the landward lagoon waters of the bay support the highest coral cover to be found in the main Hawaiian Islands (Smith et al., 1981). *Montipora capitata*, *Porites compressa*, and *Pocillopora damicornis* are found in both the outer and inner portions of the bay; however, these species are more abundant in calm lagoon waters. *Fungia scutaria* is restricted to protected lagoon reefs but is also conspicuously absent from the southeastern portion of the bay (Maragos, 1972).

*Environmental Influence on Corals*

Smith et al. (1981) concluded that circulation patterns in the bay are the primary control on the regional distribution of coral species. Corals are well known to alter colony morphology and growth rate in response to environmental conditions, although phenotypic plasiticity is variable among species (Foster, 1979; Ayre & Willis, 1988; Bruno & Edmunds, 1997; Todd, 2008; Forsman et al., 2010). Wood-Jones (1907, 1910) made the first comprehensive descriptions of growth form variations in response to physical environment in corals. Environmental conditions that are known to influence coral morphology include water flow (Vaughan, 1918), water depth, light intensity (Wood-Jones, 1910; Kawaguti, 1940; Roos, 1967; Wijsman-Best, 1972), sedimentation (Maragos et al., 1970; Roy & Smith, 1970), and storm frequency (Stoddart et al., 1966). Among tested species, Maragos (1972) observed the morphology of *Montipora capitata, Pocillopora damicornis*, and *Porites compressa* to be highly plastic in response to transplant environment, whereas *Porites lobata* and *Fungia scutaria* were least responsive to environmental conditions. Additionally, growth rates of among coral species in Kāneʻohe Bay vary in response to the local environmental conditions (Maragos, 1972). Light intensity exhibits the strongest control on coral growth rates, such that corals in shallow water environments grew signficantly faster than corals of the same species in deeper environments (Maragos, 1972). Moreover, seasonal variability in light and temperature was observed to influence growth rates suggesting that some species grew faster in winter seasons (Maragos, 1972). Also, variations in salinity positively influences growth rates in a few species (e.g. *F. scutaria*, *M. capitata*). Growth rates among species were the highest in areas where those species naturally occured in greatest abundance suggesting that the local environmental parameters also likely influence coral abundance and distribution (Maragos, 1972).

*Coral Families in Kāneʻohe Bay*

**Agariciidae**

The family Agariciidae is characterized by small star-shaped calices with radiating septo-costae that are shared by adjacent calices. Corals species of this family *Pavona varians*, *Pavona duerdeni*, *Leptoseries incrustans*, and *Leptoseries papyracae* have been documented in the bay, but recent studies indicate that *Pavona* and *Leptoseris* are not monophyletic taxa and suggest taxonomic revision is necessary in this group (Luck et al., 2013). *Pavona varians* exhibit variable encrusting and irregular mounds with contorted nodules or plate-like vertical surfaces. The coralittes are prominent and generally in rows between ridges. These corals are typically golden brown, rusty green or dark gray. *P. varians* are common on reefs at any depth inside and outside the bay, but are absent in the south lagoon (Maragos, 1972). Corals of the species *P. duerdeni* form massive colonies of thick plates with tiny calices. These corals are light gray, tan or yellow and are common in shallow waters with surge and high water flow. *L. incrustans* form small encrustations that may be smooth or lumpy dependent on light exposure. These corals have fine septa of equal wide and microscopic serrates; however, the calices are hard to distinguish. Corals of this species may be dark brown to red and are found in offshore areas of the bay in waters greater than 40 ft  (Maragos, 1972). *Leptoseris papyracea* are uncommon outside of the bay and may be found at deeper depths. These corals have thin fragile branches (1/4 inch wide) that form a clumping morphology. Also, these corals are thought to be adapted to low light conditions (Maragos, 1972).

**Acroporidae**

*Montipora* is the only genus of the family Acroporidae found in Kāneʻohe Bay. Within this genus the coral species *Montipora capitata*, *Montipora dilatata*, *Montipora fabellata*, *Montipora patula*, *Montipora verrilli*, and *Montipora studeri* can be found. *Montipora* species are classified by colony morphology and by the arrangement and size of protrusions between corallites (Veron, 2000). However, the taxonomy of *Montipora* species is uncertain due to high morphological variation in response to environmental conditions (i.e. light, sedimentation, water motion, etc.) (Todd, 2008; Forsman et al., 2010). All *Montipora* species found in the bay are simultaneous hermaphroditic spawners that release egg/sperm bundles in the summer and fall months (Richmond & Hunter, 1990). During these months, *M. dilatata*, *M. studeryi*, and *M. verrilli* shed gametes during the full moon and intermediate lunar phases (Shlesinger & Loya, 1985), while *M. capitata* releases gametes during the new moon.

*M. capitata* have high phenotypic plasticity and possess many growth forms (i.e. encrusting, plating, branching) (Maragos, 1972; Forsman et al., 2010). *Monitpora capitata* have small corallites that are well separated by coenosteum, but the septae are indistinct. The calices are surrounded by smooth surface projections (i.e. verrucae) that are irregularly spaced. The skeleton is porous and allows for its symbionts to withdraw deep into the skeleton. These corals are dark to pale brown with white verrucae and occasionally have pink, green or blue fluorescence. *Montipora capitata* cannot be characterized by a single habitat and can be found in both the outer and inner portions of the bay, but are more abundant in the calm lagoon waters. The branching and plating morphologies are common on the reef slope in the lagoon, while the encrusting form if found in high-energy environments (Maragos, 1972). Also, branching and colony size may increase with depth (Edmondson, 1929). Growth forms are unusual under natural stress (e.g. low light, high wave energy) and may be classified as separate species (see Boschma, 1954). *Montipora capitata* thrives in moderately reduced salinities and is therefore thought to be well adapted for estuarine environments such as Kāneʻohe Bay (Maragos, 1972). Also, these corals were observed to be highly sensitive to the freshwater kill events (Jokiel et al., 1993) and appeared to be resistant to the attack of *Dictyosphaeria cavernosa* (Banner & Bailey, 1970). This species may also be able to take up bacteria at higher rates in comparison to other Hawaiian corals (Franzisket, 1964). Additionally, *M. capitata* are thought to fill a similar ecological niche of the absent *Acropora* species.

*M. dilatata* are one of the rarest corals known (Forsman et al., 2010). This coral species is only found in Kāneʻohe Bay and tentatively on Maro reef in the Northwestern Hawaiian Islands. Extensive surveys in 2000 revealed only three colonies of *M. dilatata* in the bay (Maragos, 1977; Forsman et al., 2010). Colonies may exhibit any combination of morphology from encrusting to plates, knobs, and branching. The most common morphology is irregular branches up to 100 mm thick, which become flattened near their ends. *Monitpora dilatata* has no conspicuous papillae and calices are immersed between short ridges. Mitochondrial markers identified *M. dilatata* to have shared haplotypes with *M. flabellata* and *Montipora turgescens* indicating that they are either close relatives, morphological variants of the same species, or may be interbreeding (Forsman et al., 2008). It is thought that the mitochondrial genes in these corals may have not evolve rapidly enough to resolve species-level differences in skeletal characteristics, and further research is needed to resolve taxonomic uncertainty regarding these species complexes (Forsman et al., 2010).

*Montipora flabellata* are characterized by its encrusting morphology that had lilac, pink, and blue hues. These corals possess calices that are located between irregular tubercules. These corals are most abundant on shallow wave exposed reefs and are confined to outer reef areas that are free of sediment accumulation and subject to heavy surge (Maragos, 1972). Similarly, *M. patula* are characterized by an encrusting morphology that occasionally forms plates. *Montipora patula* is endemic to Hawaiʻi and exhibits a golden brown hue with white margins and bright purple polyps. The papillae are concentrated around the small and irregularly heighted corallites. Also, a ring of small rods surrounds the calices. These corals are common in a variety of environments including inside and outside of the bay and can be found on shallow exposed reefs or at more moderate to deeper depths (Maragos, 1972). *Montipora verrilli* has a thin and encrusting morphology with small corallites (~1mm diameter) that are submerged in the coenosteum. Also, these colonies have evenly spaced papillae and have a brown and blue hue. These corals can be found on reef flats in northern Kāneʻohe Bay (Maragos, 1972). Thick grayish brown clumps or sheets with thick margins characterize coral colonies of *M. studeri*. These colonies have tiny calices that are located in deep cavities that are separated by short irregular ridges. *Montipora studeri* are rarely found in shallow high wave energy environments in the bay.

**Dendrophylliidae**

The species *Tubastrea coccinea* of the diverse family, Dendrophyllidae, can be found in lower light conditions in Kāneʻohe Bay. These corals do not host *Symbiodinium spp.*; therefore, they are heterotrophic and feed at night by extending their long tentacles to catch passing zooplankton (Blomquist et al., 2006). During the day, the tentacles are retracted into deep circular calices. *T. cocinea* are hermaphroditic brooders that release planulae, which readily attach to the substratum during summer and winter months (Ayre & Resing, 1986). The young polyps of this species usually grow in total darkness (Edmondson, 1929; Richmond & Hunter, 1990). The frilly orange slug, *Phestilla melanobrachia*, is a common predator of this coral species (Faucci et al., 2007).

**Faviidae**

The large family, Faviidae, has several genera with a few species found in Kāneʻohe Bay. Coral species in the family Faviidae are characterized by encrusting or massive morphologies and large circular or hexagonal calices. Species of this family found in the bay include *Cyphastrea ocellina*, *Leptastrea bottae*, and *Leptastrea purpurea*.

*Cyphastrea ocellina* are small knobby encrusting corals that are common in shallow well mixed waters both inside and outside the bay (Maragos, 1972). These corals have elevated calices (2-3mm in diameter) and strong septal walls. This species can gain about 2mm in height annually and release brooded planulae throughout the year (Edmondson, 1929; Stimson, 1978). This species was observed to persist during the 1988 freshwater kill when other corals were eliminated (Jokiel, 1991). *Leptastrea bottae* are found in shallow well mixed waters and are more common in the outer bay (Maragos, 1972). *Leptastrea purpurea* are small usually no larger than 10 to 15cm across. These corals have a small linear increase but have a larger weight gain. The calices may varying in size with smooth ribs. Colonies of this species are brown with white centers and can be found in shallow well mixed waters and exposed reef areas (Maragos, 1972). *Leptastrea purpurea* are similar to *Leptastrea agassizi* in that both have an encrusting morphology with irregular surfaces.

**Fungiidae**

The Fungiidae family consists of solitary corals consisting of a large single polyp, with *Cycloseries vaughani* and *Fungia scutaria* found in Kāneʻohe Bay. *Cycloseries vaughani* are circular with six prominent septae with a bright orange or green fluorescent color. These corals are small (~1.5 inches) and do not survive well in shallow water due to algal overgrowth (Maragos, 1972). These corals can be found in the outer bay in deeper waters (Maragos, 1972).

*Fungia scutaria* are free-living coral species that do not attached to the substrate. These corals may have adapted its ahermatypic form to prevent smothering in sediment-rich environments (Maragos, 1972). Moreover, *F. scutaria* have the ability to remove sediment and right itself when overturned (Abe, 1937; Bosch, 1967; Rosen & Taylor, 1969). These corals are elongated in shape and have thick septae with a tall tentacular lobe lining its corallite. The septae contain unlobed teeth and the costae have rows of tiny spines. Corals vary in color but are generally brown, yellow or blue with contrasting tentacular lobes. Tentacles may be green when they are extended. The underside of the coral bears a scare from detachment as a juvenile and the parasitic snail, *Epifungium ulu*, may also be found there. *Fungia scutaria* are gonochric broadcast spawners that release very small eggs on the full moon in the summer months. These gametes undergo rapid development after fertilization into planulae larva (Krupp, 1983; Richmond & Hunter, 1990). This species is restricted to protected reefs and are completely absent in the southeastern portion of the bay (Maragos, 1972). Their distribution is highly aggregated and they are commonly found in depressions in the reef flat between coral heads where sediment is common and light intensities are reduced (Bosch, 1967). Also, *F. scutaria* show definite growth determinacy in that growth rates slow with increasing size (Bosch, 1967; Maragos, 1972). Adults may gain 5 to 6 mm annually and younger specimens may grow 4 to 6 times more rapidly when attached to older individuals from asexual reproduction  (Edmondson, 1929). Also, growth rates are stimulated with high salinity variations and inhibited with increased phosphate  (Yamazato, 1966; Maragos, 1972). Likewise, *F. scutaria* has lower respiration rates in coral in comparison to colonial hermatypes (Kawaguti, 1937). High mortality levels were observed in *F. scutaria* after the 1988 freshwater event; however, recovery of the corals was observed within one year through regeneration of new coralla on dead skeletons (Jokiel, 1991).

**Pocilloporidae**

Corals in the family Pocilloporidae have rough, solid skeletons with tiny calices. The coral species morphologies have high phenotypic plasticity in response to physical environmental factors; therefore, morphological identification may be misleading (Marti-Puig et al., 2014). Furthermore, using phylogenetic inference and morphological data have revealed cryptic speciation occurring within this family (Schmidt-Roach et al., 2014). Coral species in this family may be considered fugitive species because they inhabit reef areas that may be unsuitable for other corals (Hutchinson, 1951). These species have high density of polyps that may have adapted to increase biotic potential. Also, the high larval production may offset the disadvantages of lower photosynthetic efficiency, shorter life spans, and smaller maximum size of these species. Further, these corals have among the highest respiration rates recorded for reef corals (Yonge, 1930; Kawaguti, 1937; Franzisket, 1970). Therefore, these coral species are among the first to settle new substrates and they settle in greater numbers (Maragos, 1972). The coral species *Pocillopora eydouxi*, *Pocillopora ligulata*, *Pocillopora meandrina*, *Pocillopora molokensis*, *Pocillopora damicornis*, and possibly *Pocillopora acuta* of this family are found throughout Kāneʻohe Bay.

*Pocillopora eydouxi* have a fragile corallum with evenly size branches and evenly arranged small verrucae (Schmidt-Roach et al., 2014). These corals have rounded branch tips and are usually brown to pale in color with darker pigmentation around the oral opening of the polyp. The branches are robust and thick (2 – 4cm thick) (Schmidt-Roach et al., 2014). Also this species is similar to *P. meandrina*; however, *P. eydouxi* has styloid columella development (Schmidt-Roach et al., 2014). This species is usually found in deeper areas outside of the bay (Maragos, 1972). *Pocillopora ligulata* and *P. molokensis* are endemic to Hawaiʻi. *Pocillopora ligulata* is easily confused with *P. meandrina*, but these corals exhibit irregular, angular branches with flaring tips with loosely arranged verrucae. *Pocillopora ligulata* are restricted to shallow flats on the barrier reef and some fringing reefs with high surge (Maragos, 1972). *Pocillopora molokensis* has cylindrical branches that radiate horizontally. This species is speculated to be an ecomorph of *P. meandrina*. This species is rare and restricted to intermediate depths outside the bay (Maragos, 1972).

*Pocillopora meandrina* have lamellar and sinuous branches with evenly spaced short verrucae that form a hemispherical colony. This species shares similar morphologies as *Pocillopora verrucosa*; however, these species are very distinct genetically (Schmidt-Roach et al., 2014). *Pocillopora meandrina* are estimated to reach maximum size in 15 years and may increase linear length at 40mm per year (Edmondson, 1929; Maragos, 1972). The growth rate of *P. meandrina* is influenced by light intensity and negatively correlated with sediment cover (McVey, 1970; Maragos, 1972). Improved circulation and salinity variation also may enhance growth rates of this species. Factors controlling distribution of this species are different from those controlling growth; therefore, this species is generally restricted to high-energy areas of the bay (Maragos, 1972). However, colonies may also be found on some inner reefs where surge and circulation conditions are strong (Maragos, 1972; Smith et al., 1973).

Previously *P. damicornis* was comprised of four ecomorphs that were determined based on morphological variations attributed to local environmental conditions (Veron & Pichon, 1976; Schmidt-Roach et al., 2014). Recently, these ecomorphs were revealed as distinct species (Schmidt-Roach et al., 2012). *Pocillopora damicornis* has no true verrucae, an elongated flattened corallum and was previously referred to as the Type Y morph in the bay (Jokiel et al., 1985). The morphology is very plastic often having slender partly flattened branch that form hemispherical colonies. Colonies have even pigmentation from pink to brown. This species is found in both the outer and inner portions of the bay; however, these species are more abundance inside the calm lagoon waters. The growth of *P. damicornis* is influence by light intensity, therefore this species does not occur at depths greater than 30 ft (Maragos, 1972). Also, *P. damicornis* are estimated to reach maximum size within 6 years (Maragos, 1972). During freshwater events, *P. damicornis* was noted as the most sensitive to lowered salinity (Jokiel, 1991). Also, these corals are hermaphroditic brooder that spawns year round on the last quarter moon (Edmondson, 1946; Richmond & Hunter, 1990). This species has a rapid population turn over with high reproductive potential as they produce larvae abundantly and frequently (Harrigan, 1972; Maragos, 1972). Also, *P. damicornis* is reproductively isolated from its sister taxon, *P. acuta*.

*Pocillopora acuta* are often classified as the Type B morph of *P. damicornis* (Jokiel et al., 1985; Schmidt-Roach et al., 2014). This species is distinguished from *P. damicornis* by the pointy branch tips. In sheltered environments, elongated, fragile slender branches characterize *P. acuta*. Conversely, the corallum is compact with crowded branches in exposed environments. Also, this species is pale in color with darker pigmentation around the oral opening of the polyp (Schmidt-Roach et al., 2014). This species is found throughout the bay and in similar habitats as *P. damicornis*. *Pocillopora acuta* consistently spawn on the first quarter moon (Richmond & Jokiel, 1984).

**Poritidae**

*Porites* species of the family Poritidae are characterized by their small hexagonal calices and contribute most of the formation of the Hawaiian reefs. More than half of the corals reported in the shallow waters are species or divisions of the genus *Porites* (Edmondson, 1929; Maragos, 1972; Jokiel, 1991). These corals are important in terms of biomass and number of species inside and outside the bay. Species in this family may be more dominant due to environmental tolerances (e.g. temperature and salinity fluctuations) and the lack of *Acropora* species (Manton & Stephenson, 1935; Kinsma, 1964; Maragos, 1972). The coral species *Porites lobata*, *Porites compressa*, *Porites evermanni*, *Porites pukoensis*, *Porites duerdeni*, and *Porites brighami* are found in the bay, although colony morphology is now known to be evolutionarily plastic in this genus (Forsman et al., 2009). The species, *P. lobata*, *P. compressa*, *P. evermanni*, are gonochoric broadcast spawners (Szmant, 1986; Richmond & Hunter, 1990); however, the reproductive characteristics of other *Porites spp.* in the bay (i.e., *P. pukoensis P. duerdeni*, and *P. brighami*) are lacking (Richmond & Hunter, 1990). Reproductive trends and gonad structure are similar between *P. compressa* and *P. lobata*. Both are gonochoric broadcast spawners that exhibit lunar periodicity of gamete shedding in the summer (Stimson, 1978; Hunter, 1988). The species, *P. evermanni* spawns in late summer and early fall.

The finger-like coral, *Porites compressa* (Pōhaku puna), is considered one of the most abundant coral species and comprises ~90% of all the corals in Kāne‘ohe Bay (Edmondson, 1929; Maragos, 1972; Jokiel, 1991). This endemic species has a porous skeleton and variable morphology in which 16 different forms have been described at different levels (e.g. branching, branch fusion). Its rapid growth allows for formation of massive platforms that fuse with adjacent colonies, outcompeting other corals. *Porites compressa* is typically found in shallow sheltered reefs and lagoon environments throughout the bay. In shallow, high wave energy environments the branches of *P. compressa* are shorter. Also the branches increase in length with increasing depth and water motion (Jokiel, 1991). These corals can grow rapidly in a variety of habitats except in the most polluted parts of the bay. Also, Yamazato  (Yamazato) showed that growth rates of *P. compressa* were also restricted by increased concentrations of organic phosphate. During the *Dictyosphaeria cavernosa* invasion, this species was observed to be frequently overgrown by the native invasive alga (Banner & Bailey, 1970). Conversely, this species has been noted to be resistant to lowered salinity events through rapid regeneration of its polyps (Jokiel et al., 1993). *Porites lobata* and *P. compressa* are similar anatomically and physiologically, and at least some colonies are genetically indistinguishable (Forsman et al., 2009). Also, *P. evermanni* and *P. compressa* have been shown to merge to form a single mass but the suture lines calicles of distinct species remain independent (Edmondson, 1929).

Like *P. compressa*, the coral species *P. brighami*, *P. duerdeni*, and *P. evermanni* are endemic to Hawaiʻi. *P. brighami* is rarely located on the outer bay shallow reef areas that are exposed to heavy surge (Maragos, 1972). The colonies of this species are small and range from pinkish brown to blue or purplish gray. The calices are large and deep with pit-like sharp jagged walls. *P. duerdeni* are also rare on reefs inside Kāneʻohe Bay. Colonies of this species are large, hemispherical with spherical branch tips and are usually yellow to green in color. The calices are small and lay deep inside the outer walls to protect retracted polyps (Maragos, 1972). *Porites evermanni* are rare massive corals confined to certain areas of the barrier reef flat as well as confined to wave exposed fringing reef flats of the bay (Maragos, 1972). These massive colonies are brown or purple to gray in color with inflated squared off lobes. The calices are small with shallow smooth narrow walls. *Porites pukoensis* are rare massive corals that may form columns. This species is uncommon in the bay and confined to certain areas of the barrier reef flat and wave exposed fringing reef flats of the bay (Maragos, 1972).

*Porites lobata* is the dominant coral species on exposed reefs in Kāneʻohe Bay (Smith et al., 1973). This mounding lobe coral has small, deep calices with distinct outer walls that protect retracted polyps. Colonies are variable in color (i.e. green, yellow, tan) and shape (i.e. rounded or lobed). These corals are generally restricted to high energy areas of the bay and can be found on inner reefs with high water flow and circulation (Maragos, 1972; Smith et al., 1973). Higher growth rates of this species are correlated with areas subjected to heavy surge and shallow water with moderately lower salinities (Maragos, 1972).

**Siderastreidae**

Members of the Siderastreidae family have a flower like pattern that is created by enlarged septa and sinuous lines that connect adjacent calices. The species *Psammocora stellate* and *Psammocora verrilli* are found in Kāneʻohe Bay. *Psammocora stellata* are tan, brown and rarely pink with very small calices that are slightly depressed with a flower-like appearance. These corals are common on shallow reef flats of shallow depths outside the bay in well mixed water (Maragos, 1972). *Psammocora verrilli* are endemic to Hawaiʻi and has an encrusting morphology. The corallites are in individual shallow depressions with thin septo-costae and septae fused into groups. The columellae are well developed in this species. This species is rare in the outer bay.

**References**

Abe N. 1937. Ecological survey of Iwayama Bay, Palao. *Trop Biol Stn Std* 1:217-324.

American-European Congress of Ophthalmic Surgery A. 1981. Oahu coral reef inventory. Hawaii Coral Reef Inventory: island of Oahu. U.S. Army Engineer Corp. Engineers, Pacific Ocean Division. p 70-159.

Ayre D, and Willis B. 1988. Population structure in the coral Pavona cactus: clonal genotypes show little phenotypic plasticity. *Marine Biology* 99:495-505.

Ayre DJ, and Resing JM. 1986. Sexual and asexual production of planulae in reef corals. *Marine Biology* 90:187-190.

Bahr KD, Rodgers KS, and Jokiel PL. in press 2015. Recent freshwater reef kill event in Kāne‘ohe Bay, Hawai‘i. *Reef Encounter: Scientific Note*.

Banner AH. 1974. Kaneohe Bay, Hawaii: urban pollution and a coral reef ecosystem. In: Cameron AMea, editor. Proceedings of the Second International Symposium on Coral Reefs. Brisbane Australia. p 685-702.

Banner AH, and Bailey JH. 1970. The effects of urban pollution upon a coral reef system: A preliminary report.

Bathen KH. 1968. A descriptive study of the physical oceanography of Kāne‘ohe Bay , Oahu, Hawaii. Honolulu (HI). Hawai’i Institute of Marine Biology, University of Hawai’i.

Bird CE, Franklin EC, Smith CM, and Toonen RJ. 2013. Between tide and wave marks: a unifying model of physical zonation on littoral shores. *PeerJ* 1:e154.

Blomquist CH, Lima PH, Tarrant AM, Atkinson MJ, and Atkinson S. 2006. 17ß-Hydroxysteroid dehydrogenase (17ß-HSD) in scleractinian corals and zooxanthellae. *Comparative Biochemistry and Physiology, Part B* 143:397-403.

Bosch HF. 1967. Growth rate of *Fungia scutaria* in Kaneohe Bay, Oahu M.S. University of Hawaii.

Boschma H. 1954. On some specimens of the coral Montipora verrucosa (Lamarck) from the Hawaiian Islands, formerly described as separate species. Ned Acad Wet Amsterdam Proc Ser C, 57, 151-158.

Bruno JF, and Edmunds PJ. 1997. Clonal variation for phenotypic plasticity in the coral Madracis mirabilis. *Ecology* 78:2177-2190.

Cox DC, Fan PF, Chave KE, Clutter RI, Gundersen KR, Burbank NC, Lau LS, Davidson JR, Helfrich P, Gray BL, Yamauchi HA, Afifi HA, Rutherford F, Young RHF, and Morphew KL. 1973. Estuarine pollution in the State of Hawaii. Kaneohe Bay Study. Water Resources Research Center Technical Report University of Hawaii. p 444.

Demopoulos AW, and Smith CR. 2010. Invasive mangroves alter macrofaunal community structure and facilitate opportunistic exotics. *Marine Ecology Progress Series* 404:51-67.

Devaney DM, Kelly M, Lee PJ, and Motteler LS. 1982. *Kaneohe: a history of change (1778-1950)*: Bishop Mueseum.

Drupp PS, DeCarlo EH, Mackenzie FT, Bienfang P, and Sabine CL. 2011. Nutrient inputs, phytoplankton response and CO2 variations in a semi-enclosed subtropical embayment, Kāne‘ohe Bay , Hawaii. *Aquatic Geochemistry* 17:473-498.

Edmondson CH. 1929. *Growth of Hawaiian Corals*. Honolulu HI: Bishop Museum

Edmondson CH. 1946. Behavior of coral planulae under altered saline and thermal conditions. *Occ Pap Bishop Museum* 18:283-304.

Fagan KE, and Mackenzie FT. 2007. Air-sea CO2 exchange in a subtropical estuarine-coral reef system, Kāne‘ohe Bay , Oahu, Hawaii. *Marine Chemistry* 106:174-191.

Faucci A, Toonen RJ, and Hadfield MG. 2007. Host shift and speciation in a coral-feeding nudibranch. *Proceedings of the Royal Society B: Biological Sciences* 274:111-119.

Fautin D, Dalton P, Incze LS, Leong J-AC, Pautzke C, Rosenberg A, Sandifer P, Sedberry G, Tunnell Jr JW, and Abbott I. 2010. An overview of marine biodiversity in United States waters. *PLoS One* 5:e11914.

Fletcher CH, and Jones AT. 1997. Sea-level highstand recorded in Holocene shoreline deposits on Oahu, Hawaii. *Oceanographic Literature Review* 44.

Forsman ZH, Barshis DJ, Hunter CL, and Toonen RJ. 2009. Shape-shifting corals: molecular markers show morphology is evolutionarily plastic in Porites. *BMC evolutionary biology* 9:45.

Forsman ZH, Concepcion GT, Haverkort RD, Maragos JE, and Toonen RJ. 2008. Genetic and Morphological Characterization of a Coral Species of Concern Montipora dilatata in Kaneohe Bay, Oahu, Hawaii. Pacific Islands Region Species of Concern 2008 Project Report.

Forsman ZH, Concepcion GT, Haverkort RD, Shaw RW, Maragos JE, and Toonen RJ. 2010. Ecomorph or endangered coral? DNA and microstructure reveal hawaiian species complexes: Montipora dilatata/flabellata/turgescens & M. patula/verrilli. *PLoS One* 5:e15021.

Foster AB. 1979. Phenotypic plasticity in the reef corals *Montastraea annularis* (Ellis & Solander) and *Siderastrea sidereal* (Ellis & Solander). . *Journal of Experimental Marine Biology and Ecology* 39:25-54.

Gattuso JP, Frankignoulle M, Bourge I, Romaine S, and Buddemeier RW. 1998. Effect of calcium carbonate saturation of seawater on coral calcification. *Global Planetary Change* 18:37-46.

Giambelluca TW, Nullet MA, and Schroeder TA. 1986. *Rainfall Atlas of Hawaii*: Department of Land and Natural Resources, State of Hawaii.

Grigg RW. 1994. Community structure, succession and development of coral reefs in Hawaii. *A Natural History of the Hawaiian Islands: Selected Readings II*:196.

Harrigan JF. 1972. The planula larva of *Pocillopora damicornis*: Lunar periodicity of swarming and substraum selection behavior Ph. D. University of Hawaii.

Hearn C, and Atkinson MJ. 2000. Effects of sea-level rise on the hydrodynamics of a coral reef lagoon: Kaneohe Bay, Hawaii. In: Noye H, ed. *Sea-level changes and their effects*. Singapore: World Scientific Publishing Pte. Ltd., 25-48.

Holthus P. 1986. Coral reef communities of Kaneohe Bay: an overview. . In: Jokiel PL, Richmond RH, and Rogers RA, eds. *Coral reef population biology*. Honolulu HI: Sea Grant Cooperative Report

Hoover W. 2004. Oahu beaches, streams spoiled by sewage spills. Honolulu Advertiser. Honolulu.

Hunter CL. 1988. Genotypic diversity and population structure of the Hawaiian reef coral, *Porites compressa* Ph. D. University of Hawaii.

Hunter CL, and Evans CW. 1995. Coral reefs in Kaneohe Bay, Hawaii: two centuries of western influence and two decades of data. *Bulletin of Marine Science* 57:501-515.

Hutchinson GE. 1951. Copepodology for the ornithologist. *Ecology* 32:471-477.

Jokiel PL. 1991. *Jokiel's Illustrated Scientific Guide to Kaneohe Bay.* .

Jokiel PL. 2008. Biology and ecological functioning of coral reefs in the main Hawaiian Islands. In: Riegl B, and Dodge RE, eds. *Corals Reefs of the USA*. Netherlands: Springer, 489-517.

Jokiel PL, Hunter CL, Taguchi S, and Watarai L. 1993. Ecological impact of a fresh-water “reef kill” in Kaneohe Bay, Oahu, Hawaii. *Coral Reefs* 12:177-184.

Jokiel PL, Ito RY, and M. LP. 1985. Night irradiance and synchronization of lunar spawning of larvae in the reef coral *Pocillopora damicornis* (Linnaeus). *Marine Biology* 88:167-174.

Jury C, Thomas F, Atkinson M, and Toonen R. 2013. Buffer Capacity, Ecosystem Feedbacks, and Seawater Chemistry under Global Change. *Water* 5:1303-1325.

Kaneohe Bay Information System K. 2014. Hawaii Institute of Marine Biology.

Kawaguti S. 1937. On the physiology of reef corals. I. On the oxygen exchange of reef corals. *Trop Biol Stn Std* 1:187-198.

Kawaguti S. 1940. Materials for the study of reef-building corals. *Japan Society Promotion Science Resources* 2:159-169.

Klim DG. 1969. Interactions between sea water and coral reefs in Kaneohe Bay, Oahu, Hawaii Ph. D. University of Hawaii.

Krauss KW, and Allen JA. 2003. Influences of salinity and shade on seedling photosynthesis and growth of two mangrove species, Rhizophora mangle and Bruguiera sexangula introduced to Hawaii. *Aquatic Botany* 77:311-324.

Krupp DA. 1983. Sexual reproduction and early development of the solitary coral, *Fungia scutaria* (Anthozoa: Scleractinia). *Coral Reefs* 2:159-164.

Lowe RJ, Falter JL, Monismith SG, and Atkinson MJ. 2009. Wave-driven circulation of a coastal reef–lagoon system. *Journal of Physical Oceanography* 39:869-889.

Luck DG, Forsman ZH, Toonen RJ, Leicht SJ, and Kahng SE. 2013. Polyphyly and hidden species among Hawaiʻi’s dominant mesophotic coral genera, Leptoseris and Pavona (Scleractinia: Agariciidae). *PeerJ*, *1*, e132.

Manton SM, and Stephenson TA. 1935. Ecological surveys of coral reefs. British Museum.

Maragos JE. 1972. A Study of the Ecology of Hawaiian Reef Corals Ph. D. University of Hawaii.

Maragos JE. 1977. Order Scleractinia. In: Devaney DM, and Eldredge LG, eds. *Reef and shore fauna of Hawai’i Section 1: Protozoa through Ctenophora*. Honolulu HI: Bishop Museum Press, 158-241.

Maragos JE, and Chave KE. 1973. Stress and interference of man in the bay. . In: Smith SV, Chave KE, and Kam DO, eds. *Atlas of Kaneohe Bay: a reef ecosystem under stress*. Sea Grant College Programs, University of Hawaii, 119-124.

Maragos JE, Roy KJ, and Smith SV. 1970. Corals collected at Fanning Atoll 1968-1970. *Hawaii Institute Geophysics*:77-84.

Marti-Puig P, Forsman ZH, Haverkort-Yeh RD, Knapp IS, Maragos JE, and Toonen RJ. 2014. Extreme phenotypic polymorphism in the coral genus Pocillopora; micro-morphology corresponds to mitochondrial groups, while colony morphology does not. *Bulletin of Marine Science* 90:211-231.

Massaro R, DeCarlo EH, Drupp PS, Mackenzie FT, Jones S, Shamberger KEF, Sabine CL, and Freely R. 2012. Multiple factors driving variability of CO2 exchange between the ocean and atmosphere in a tropical coral reef environment. *Aquatic Geochemistry* 18:357-387.

McVey J. 1970. Fishery ecology of the Pokai artifical reef Ph. D. University of Hawaii.

Ostrander CE, McManus MA, DeCarlo EH, and Mackenzie FT. 2007. Temporal and Spatial Variability of Freshwater Plumes in a Semienclosed Estuarine–Bay System. *Estuaries and Coasts* 31:192-203.

Paquay FS, Mackenzie FT, and Borges AV. 2007. Carbon dioxide dynamics in rivers and coastal waters of the‘‘big island’’ of Hawaii, USA, during baseline and heavy rain conditions. *Aquatic Geochemistry* 13:1-18.

Richmond RH, and Hunter CL. 1990. Reproduction and recruitment of corals: Comparisons among the Caribbean, the Tropical Pacific, and the Red Sea. *Marine ecology progress series* 60:185-203.

Richmond RH, and Jokiel PL. 1984. Lunar periodicity in larva release in reef coral *Pocillopora damicornis* at Enewetak and Hawaii. *Bulletin of Marine Science* 34:280-287.

Ringuet S, and Mackenzie FT. 2005. Controls on nutrient and phytoplankton dynamics during normal flow and storm runoff conditions, southern Kaneohe Bay, Hawaii. *Estuaries* 28:327-337.

Roos PJ. 1967. *Growth and occurrence of the reef coral Porites astreodies Lamarck in relation to submarine radiance distribution.* Drukkerij Elinkwijk, Utrecht.

Rosen BR, and Taylor JD. 1969. Reef coral from Aldabra. A new mode of reproduction. *Science* 166:119-121.

Roy KJ. 1970. Change in bathymetric configuration, Kaneohe Bay, Oahu. 1882-1969. Hawaii Institute Geophysics Report. Honolulu: University of Hawaii. p 226.

Roy KJ, and Smith SV. 1970. Note on the growth of Pocillopora ligulata. *Hawaii Institute Geophysics* 70-23:179-189.

Schmidt-Roach S, Lundgren P, Miller K, Gerlach G, Noreen A, and Andreakis N. 2012. Assessing hidden species diversity in the coral Pocillopora damicornis from Eastern Australia. *Coral Reefs* 32:1-12.

Schmidt-Roach S, Miller KJ, Lundgren P, and Andreakis N. 2014. With eyes wide open: a revision of species within and closely related to thePocillopora damicornisspecies complex (Scleractinia; Pocilloporidae) using morphology and genetics. *Zoological Journal of the Linnean Society* 170:1-33.

Shamberger KEF, Feely RA, Sabine CL, Atkinson MJ, DeCarlo EH, Mackenzie FT, Drupp PS, and Butterfield DA. 2011. Calcification and organic production on a Hawaiian coral reef. *Marine Chemistry* 127:64-75.

Shlesinger Y, and Loya Y. 1985. Coral community reproductive patterns: Red Sea versus the Great Barrier Reef. *Science* 228:1333-1335.

Silbiger NJ, Guadayol O, Thomas FI, and Donahue MJ. 2014. Reefs shift from net accretion to net erosion along a natural environmental gradient. *Marine Ecology Progress Series* 515:33-44.

Siple MC, and Donahue MJ. 2013. Invasive mangrove removal and recovery: Food web effects across a chronosequence. . *Journal of Experimental Marine Biology and Ecology* 448:128-135.

Smith SV, Chave KE, and Kam DO. 1973. *Atlas of Kaneohe Bay: a reef ecosystem under stress*. Honolulu HI: University of Hawaii Sea Grant.

Smith SV, Kimmerer WJ, Laws EA, Brock RE, and Walsh TW. 1981. Kaneohe Bay sewage diversion experiment: perspectives on ecosystem responses to nutritional perturbation. *Pacific Science* 35:279-395.

Stimson J. 1978. Mode and timing of repoduction in common hermatypic corals of Hawaii and Enewetak. *Marine Biology* 48:173-184.

Stimson J, Larned S, and Conklin E. 2001. Effects of herbivory, nutrient levels, and introduced algae on the distribution and abundance of the invasive macroalga Dictyosphaeria cavernosa in Kaneohe Bay, Hawaii. *Coral Reefs* 19:343-357.

Stoddart DR, Davies PS, and Keith A. 1966. Geomorphology of Addu Atoll. *Atoll Research Bulletin* 116:13-41.

Szmant AM. 1986. Reproductive ecology of Carribean reef corals. *Coral Reefs* 5:43-53.

Taguchi S, and Laws EA. 1987. Patterns and causes of temporal variability in the physiological condition of the phytoplankton community in Kaneohe Bay, Hawaii. *Journal of Plankton Research* 9:1143-1157.

Takasaki KJ, Hirashima GT, and Lubke ER. 1969. Water resources of windward Oahu, Hawaii. US Geological Survey Water Supply Paper p119.

Todd P. 2008. Morphological plasticity in scleractinian corals. *Biological Reviews* 83:315-337.

Vaughan TW. 1918. Some shoal-water corfals from Murray Island, Cocos-Keeling Islands, and Fanning Island. *Publs Carnegie Instn Wash*:213-234.

Veron JEN. 2000. *Corals of the world*. Townsville: Australian Institute of Marine Science.

Veron JEN, and Pichon M. 1976. Scleractinia of Eastern Australia. *Monograph Series of the Australian Institute for Marine Science I*.

Vroom PS. 2011. “Coral dominance”: a dangerous ecosystem misnomer? *Journal of Marine Biology*.

Vroom PS, and Braun CL. 2010. Benthic composition of a healthy subtropical reef: baseline species-level cover, with an emphasis on algae, in the Northwestern Hawaiian Islands. *PLoS One* 5:e9733.

Wijsman-Best M. 1972. Systematics and ecology of New Caledonian Faviinae (Coelenterata-Scleractinia). *Bijdr Diek* 42:1-95.

Wollast R. 2002. Continental margins—review of geochemical settings. In: Wefer G, Billett D, Hebbeln D, Jorgensen BB, and von Weesing T, eds. *Ocean margin systems* Springer, 15-31.

Wood-Jones F. 1907. On the growth forms and supposed species in corals. *Proceedings in the Zoological Society London*:518-556.

Wood-Jones F. 1910. *Corals and Atolls*. London.

Yamazato K. 1966. Calcification in a solitary coral, *Fungia scutaria* (Lamarck) in relation to environmental factors Ph. D. University of Hawaii.

Yonge CM. 1930. Studies on the physiology of corals. I. Feeding mechanisms and food. Grt. Barrier Reef Exped., 1928-29, Sci. Repts. 1: 15-57.
